# Geographic filling delay of the choriocapillaris in the region of dilated asymmetric vortex veins in central serous chorioretinopathy

**DOI:** 10.1371/journal.pone.0206646

**Published:** 2018-11-09

**Authors:** Shoji Kishi, Hidetaka Matsumoto, Shozo Sonoda, Takashi Hiroe, Taiji Sakamoto, Hideo Akiyama

**Affiliations:** 1 Maebashi Central Eye Clinic, Maebashi, Japan; 2 Department of Ophthalmology, Gunma University Graduate School of Medicine, Maebashi, Japan; 3 Department of Ophthalmology, Kagoshima University Graduate School of Medical and Dental Sciences, Kagoshima, Japan; Massachusetts Eye & Ear Infirmary, Harvard Medical School, UNITED STATES

## Abstract

**Purpose:**

To investigate the correlation between geographic filling delays in the choriocapillaris using indocyanine green angiography (ICGA) images and dilated vortex veins in central serous chorioretinopathy (CSC).

**Design:**

Observational case series.

**Participants:**

Thirty-two eyes of 32 patients, 21 with acute and 11 with chronic CSC.

**Methods:**

Digital ICGA and fluorescein angiography (FA), with videoangiography, and swept-source optical coherence tomography (SS-OCT) for B-scan and en-face choroidal imaging were performed. Overlapping of the filling delay areas in the choriocapillaris in the early-phase ICGA images and the region of dilated vortex veins in the en-face images were analyzed. The consistency of both areas was graded as follows. Grade 3: filling delay area is entirely involved in the dilated vortex vein region. Grade 2: 50% or more of filling delay area overlaps with the dilated vortex vein region. Grade 1: less than 50% of filling delay area overlaps with the dilated vortex vein region. Grade 0: no tendency for overlapping of two areas. We evaluated the asymmetry of upper and lower vortex veins in en-face images of the Haller layer. Using the binarization method, we quantified the luminal and stromal areas of the choroid. The ratios of the Haller layer area and luminal areas in the Haller layer to total choroidal area were examined.

**Results:**

The consistency of overlapping of the two areas was grade 2.62 ± 0.49 in acute CSC and grade 1.55 ± 0.78 in chronic CSC (p = 0.0005). Asymmetry of upper and lower vortex veins was seen in 17 of 22 eyes (81%) with acute CSC and 6 of 11 eyes (54.5%) with chronic CSC (p = 0.114). Central choroidal thickness was 411 ± 79 μm in acute CSC and 326 ± 64 μm in chronic CSC (p = 0.004). In the posterior fundus with a 4500 μm diameter, the ratio of the Haller layer area to total choroidal area was 63.7 ± 8.6% in acute CSC and 57.1 ± 7.9% in chronic CSC (p = 0.047). The ratio of the luminal area in the Haller layer area to total choroidal area was 46.9 ± 7.6% in acute CSC and 40.0 ± 6.9% in chronic CSC (p = 0.014)

**Conclusion:**

Filling delay areas in the choriocapillaris and dilated vortex vein regions showed marked overlapping in acute CSC. Increased choroidal thickness was attributed to dilated vortex veins. These findings suggest that the blood flow into the choriocapillaris is delayed as a result of congestion of the dominant vortex veins that supply this geographic area. CSC may be a disease characterized by vortex vein congestion that develops in eyes with asymmetric vortex veins.

## Introduction

Central serous chorioretinopathy (CSC) is part of the spectrum of pachychoroid diseases that include CSC, pachychoroid pigment epitheliopathy [1], pachychoroid neovasculopathy [2], and polypoidal choroidal vasculopathy [3]. With the introduction of enhanced depth imaging[4], increased choroidal thickness [5–7], dilated outer choroidal vessels [8,9] and increased thickness of the Haller layer [10] have been identified in CSC and other pachychoroid diseases [1–3,11].

The vortex veins are choroidal drainage routes that pass through the sclera. Horizontal and vertical watersheds divide the choroidal vasculature into four quadrants. Each vortex vein independently serves each quadrant [12]. The routes of the vortex veins vary greatly [13–15]. Using en-face choroidal imaging, we investigated the patterns of the vortex veins in the posterior fundus in eyes with CSC and normal control eyes [16]. The upper and lower vortex veins were symmetrically distributed in 62% and asymmetrically distributed in 38% of normal eyes. In eyes with CSC, the upper and lower vortex veins were consistently asymmetric [16]. Dominant vortex veins supplied the entire macula or the posterior pole and showed marked dilation from their distal ends. These dilated vortex veins corresponded to the dilated outer choroidal vessels seen on B-scans of swept-source optical coherence tomography (SS-OCT) [16]. We hypothesized that asymmetric vortex veins are a factor predisposing to the development of CSC [16]. Congestion of the dominant vortex vein appears to cause CSC. Using ultra-widefield indocyanine green angiography (ICGA), Pang et al. reported all of the features of the dominant vortex veins in CSC [17]. The dominant vortex veins involved the entire posterior pole and were dilated from the distal end to the ampulla.

On ICGA, a geographic filling delay of the choriocapillaris is a well-known phenomenon in CSC [18–20]. However, because the manner in which the filling delay correlates with the congestion of the vortex veins has yet to be revealed, we attempted to clarify the correlation between these two factors in CSC.

## Methods

This study was conducted according to the Declaration of Helsinki; the institutional review board and the ethics committee of Gunma University Graduate School of Medicine approved the study. Each subject provided written informed consent before any study procedures or examinations were performed.

### Participants

We retrospectively reviewed the medical records of all patients diagnosed with CSC at Gunma University Hospital between January 2017 and January 2018. The diagnosis of CSC was made if serous retinal detachment was seen in the macular area by fundus ophthalmoscopy and SS-OCT, dye leakage within the serous retinal detachment on FA and choroidal vascular hyperpermeability on ICGA. We excluded the eyes with serous retinal detachment secondary to age-related macular degeneration, uveitis, retinal vascular diseases or diabetic retinopathy. Eligible patients met the following inclusion criteria: good-quality early-phase ICGA, B-scan OCT images, and en-face images of the choroidal vessels. If both eyes were eligible, the right eye was selected.

In this study, we defined acute CSC as that occurring within 6 months of symptom onset and chronic CSC as that occurring more than 6 months after symptom onset.

### Multimodal imaging methods

All patients underwent a comprehensive ophthalmologic examination, including best-corrected visual acuity (BCVA) and intraocular pressure measurement, indirect ophthalmoscopy, slit-lamp biomicroscopy with a noncontact lens, color fundus photography, fundus autofluorescence (FAF), SS-OCT, FA, and ICGA. FA, ICGA and FAF were acquired using a confocal scanning laser ophthalmoscope (HRA2; Heidelberg Engineering, Heidelberg, Germany) with the field of view set to 30°×30°, which included an approximately 9×9 mm area centered on the fovea in the fundus. We documented the early-phase ICGA and FA findings during the first 30 seconds by digital videoangiography starting from the entrance of the dye into the fundus and then obtained the late-phase angiograms. The SS-OCT images were acquired with SS-OCT (DRI OCT-1 Atlantis, Topcon, Tokyo, Japan). We obtained B-scan images of the horizontal and vertical line scans (12 mm) through the fovea. The volume data were obtained with a raster scan protocol of 512 (horizontal) × 128 (vertical) B-scans in 0.8 second, which covered a 9 × 12-mm area centered on the fovea. The en-face images were obtained as coronal slices from a 3-dimensional dataset using En View software (Topcon), which flattened the images at the level of Bruch’s membrane. Each en-face image, which included the depth from Bruch’s membrane to the chorioscleral interface, was an average of three consecutive images.

### Image analysis

**Fig 1:**
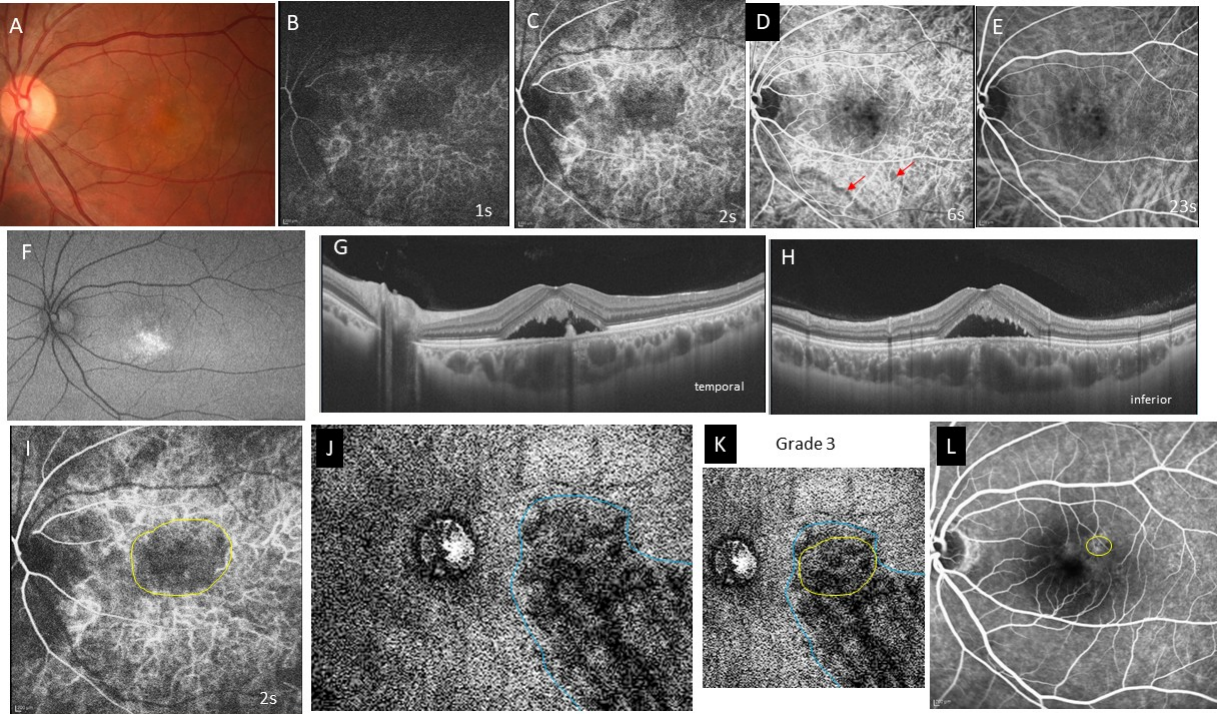
Case 1, a 36-year-old man, had acute central serous chorioretinopathy in the left eye of. The best-corrected visual acuity was 0.7 with -3.5 diopters of myopia. **A,** Color fundus photograph shows serous retinal detachment in the macula. **B-E,** Indocyanine green angiography (ICGA) images at 1, 2, 6, and 23 seconds (s) from the entrance of dye into the fundus. A choroidal filling delay is seen at the arteriolar (B) and choriocapillaris phases (C). The macula remains dark during the venous (D) and late phases (E). **D,** A filling delay is seen in the inferior vortex vein (red arrows) at 6s. **F,** Fundus autofluorescence shows accumulation of hyperfluorescent material in the inferior subretinal space. **G,** Horizontal swept-source optical coherence tomography B-scan image shows the pachychoroid (central choroidal thickness; 471μm) with markedly dilated vortex veins and a serous retinal detachment with an elongated photoreceptor outer segment. **H,** Vertical B-scan shows findings similar to those of the horizontal B-scan. **I,** ICGA image at 2s shows filling delay area in the choriocapillaris outlined in yellow. **J,** An en-face image of the choroid at the plane containing the dominant vortex vein, the region of which is outlined in blue. **K,** Superimposition of the filling delay area (yellow line) onto the dilated vortex vein region (blue line) is shown. Overlapping of the two areas is grade 3. **L,** Late-phase fluorescein angiography image. The leakage site within the yellow circle is at the distal end of the dilated vortex vein in J.

**Fig 2:**
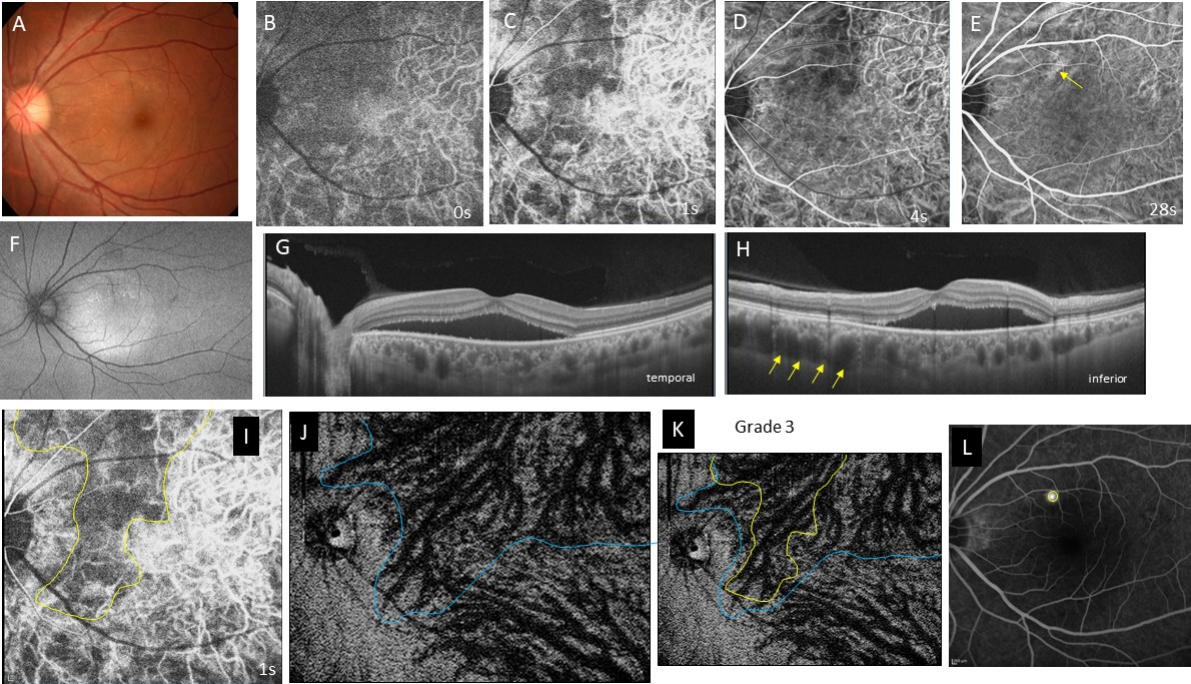
Case 2, a 39-year-old man, had acute central serous choriocapillaris in the left eye. The best-corrected visual acuity is 1.2 with emmetropia. **A,** Color fundus photograph shows a serous retinal detachment in the posterior pole. **B-E,** Indocyanine green angiography images at 0 (B), 1 (C), 4 (D), and 28 seconds (s) (E) from the entrance of the dye into the fundus. **B,** During the arteriolar phase, a filling delay is seen in the nasal half of the posterior fundus. **C,** During the choriocapillaris phase, a vertical filling delay is seen nasal to the fovea. **D,** The superior half of the zone remains dark even in the venous phase. **E,** The dark area becomes obscured. Hyperfluorescence is seen in a dilated vortex vein (yellow arrow) that corresponds to the leakage point on a fluorescein angiography image, as indicated by the yellow circle in L. **F,** Fundus autofluorescence shows increased fluorescence in the area of the serous retinal detachment. **G, H,** Swept-source optical coherence tomography B-scan image, in the horizontal (G) and vertical sections (H), shows a serous retinal detachment with pachychoroid (central choroidal thickness; 459μm). Marked dilatation of the vortex veins (yellow arrows) is seen superior to the macula in H. **I,** Geographic area of the filling delay at the choriocapillaris phase in C is outlined in yellow. **J,** An en-face image of the choroid at the plane containing the dominant vortex vein, the region of which is outlined in blue. **K,** Superimposition of the filling delay area (yellow line) on the dilated vortex vein region (blue line) is shown. Overlapping of the two areas is grade 3. **L,** Late-phase fluorescein angiography. The leakage site in the yellow circle is on the dilated vortex vein in J.

**Fig 3:**
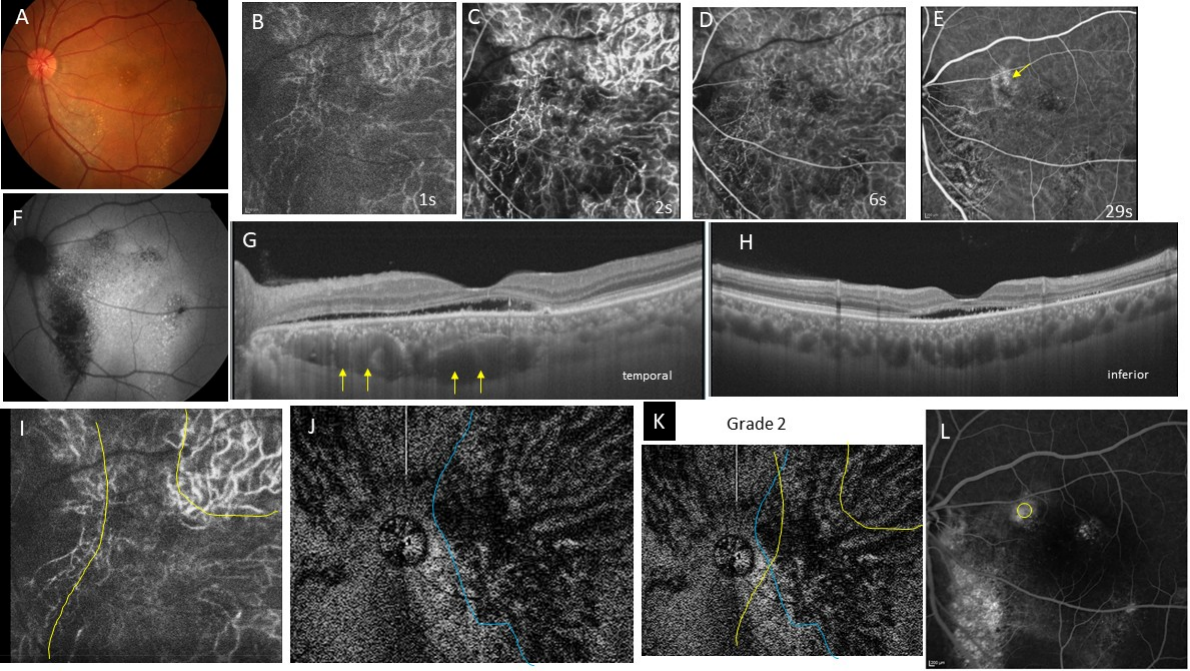
Case 3, 48-year-old man, had chronic central serous chorioretinopathy in the left eye. The best-corrected visual acuity is 0.5 with +1.25 diopters of hyperopia. **A,** Color fundus photograph shows yellow precipitates at the macula and zonal atrophy with yellowish spots corresponding to the old descending track of subretinal fluid. **B-E,** Indocyanine green angiography (ICGA) images at 1 (B), 2 (C), 6 D), and 29 seconds (s) (E) from entrance of the dye into the fundus. **B,** Arteriolar phase. **C,** A large filling delay is seen in the vertical zone between the disc and macula and the horizontal zone including the macula and its temporal vicinity. **D,** The filling delay in C remains dark during the venous phase. **E,** The filling delay is obscured. Hyperfluorescence (yellow arrow) is seen at the dilated vortex vein, which corresponds to the leakage on the fluorescein angiography image (L). **F,** Fundus autofluorescence shows hyperfluorescent dots and a focal area of hypofluorescence in the area of the old lesion. **G, H,** Horizontal (G) and vertical (H) detachment with marked dilatation of the vortex vein (yellow arrows in G) in the macula and nasally. The choroid shows pachychoroid (central choroidal thickness; 423 μm). **I,** ICGA image of the choriocapillaris phase at 1.5s. The filling delay area is outlined in yellow. **J,** An en-face image of the choroid at the plane containing the dilated dominant vortex vein. The dilated vortex vein region is outlined in blue. **K,** Superimposition of the filling delay area (yellow line) on the dilated vortex vein region (blue line) is shown. The extents of both areas were ill defined but approximately overlapped. Overlapping is grade 2. **L,** Late-phase fluorescein angiography. The yellow circle indicates the leakage site, which corresponds to the distal end of the dilated vortex vein (J). Hyperfluorescence is seen in the area of the descending tract due to a window defect in the atrophic retinal pigment epithelium.

**Fig 4:**
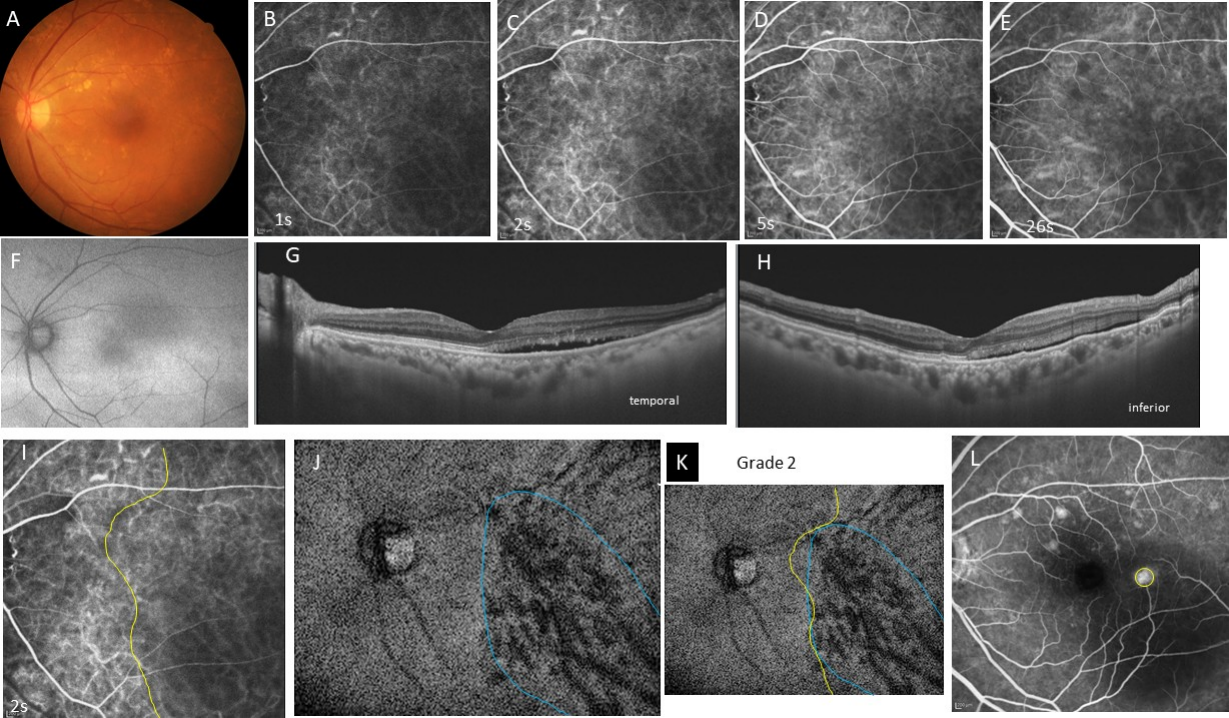
Case 4, a 77-year-old woman, had chronic central serous chorioretinopathy in the left eye. The best-corrected visual acuity is 0.6 with +2.75 diopters of hyperopia. **A,** Color fundus photograph shows diffuse drusen in the posterior pole. **B-E,** Indocyanine green angiography images at 1 (B), 2 (C), 5 (D), and 26 seconds (s) (E) from entrance of the dye into the fundus. A filling delay is seen in the inferotemporal quadrant at the arteriolar (B) and choriocapillaris phases (C). This area remains dark in the venous (D) and late phases (E). **F,** Fundus autofluorescence image shows weak hyperfluorescence in the inferotemporal quadrant. **G, H,** Horizontal (G) and vertical (H) swept-source optical coherence tomography B-scans show a small serous retinal detachment temporal (G) and inferior to the fovea (H). The choroid has no pachychoroid (central choroidal thickness; 357μm) but the vortex veins are moderately dilated. **I,** Filling delay area in the choriocapillaris in C is outlined in yellow. **J,** An en-face image of the choroid at the plane containing the dominant vortex veins, the region of which is outlined in blue. **K,** Superimposition of the filling delay area (yellow line) on the dilated vortex vein region (blue line) is shown. Extent of the filling delay area is ill defined. The area is larger than the dilated vortex vein region. Overlapping of the two areas is grade 2. **L,** Late-phase fluorescein angiography. The yellow circle indicates the site of dye leakage corresponding to the dilated vortex vein in J. The hyperfluorescent spots indicate the presence of multiple drusen.

We compared the filling delay areas seen in the choriocapillaris on the early phase ICGA images and the region of the dilated vortex veins seen in the en-face images. Still images of ICGA during the choriocapillaris perfusion phase were captured from digital videoangiography. We manually demarcated the filling delay areas on the ICGA images with yellow lines (Figs 1I, 2I, 3I and 4I). On the en-face image of the outer Haller layer, the dilated vortex vein region was manually outlined in blue (Figs 1J, 2J, 3J and 4J). After the location and magnification of both images had been adjusted, the contour of filling delay areas was superimposed on the en-face images of the outer Haller layer, where the dilated vortex vein region was outlined in blue (Figs 1K, 2K, 3K and 4K). We graded the consistency of overlapping between the filling delay areas and dilated vortex vein regions, as follows. Grade 3: Filling delay area is entirely involved in the dilated vortex vein region. Grade 2: 50% or more of the filling delay area overlaps with the dilated vortex vein region. Grade 1: Less than 50% of filling delay area overlaps with the dilated vortex vein region. Grade 0: no tendency for overlapping of the two areas.

We also evaluated the asymmetry of the upper and lower vortex veins in en-face images of the outer Haller layer. If dilated vortex veins extended over the horizontal line through the fovea and they involved the entire macula or the posterior pole, we considered asymmetry to be present, i.e. the determination was “yes”. If no distinct differences in extent and vascular dilatation were seen in either upper or lower vortex veins, asymmetry was considered to be absent, i.e. the determination was “no”. The grading of consistency and asymmetry in each case was determined by two authors reaching agreement (S.K, H.M).

Central choroidal thickness was defined as the distance between Bruch’s membrane and the chorioscleral interface at the fovea. Subfoveal choroidal thickness was measured manually on SS-OCT images using the built-in caliper. Horizontal and vertical scans including the foveal center were used, and the values were averaged.

To assess the contribution of vortex veins to increased choroidal thickness, we quantified the luminal and stromal areas of the choroid employing the binarization technique [21]. The B-scan images of the posterior fundus with a 4500μm diameter centered on the fovea were binarized. The ratios of the Haller layer area and luminal areas in the Haller layer to total choroidal area were calculated in horizontal and vertical B-scans, and the values were averaged. The Haller layer is defined the area between the chorioscleral interface and the innermost border of the large outer choroidal vessel.

We studied the relationship between the sites of dye leakage on FA images and en-face images of vortex veins.

### Statistical analysis

The data analyses were performed using Excel 2016 (Microsoft, Redmond, WA, USA) with add-in software Statcel 4 [22]. All values are expressed as means ± standard deviations. *P* values less than 0.05 were considered significant.

## Results

Thirty-two patients met the study eligibility criteria. Twenty-one patients were diagnosed with acute CSC and 11 were diagnosed with chronic CSC. Two patients were taking corticosteroids, one with chronic CSC for interstitial pneumonia and one with acute CSC for Crohn’s disease. Four patients, 3 with chronic CSC and one with acute CSC, developed recurrent CSC. All patients were Japanese, and most were male. Patients with acute CSC were significantly younger than those with chronic CSC (46.8 ± 8.9 years vs. 61.8 ± 13.3 years; p = 0.005). There was no significant between-group difference in BCVA. Patients with acute CSC were more myopic than those with chronic CSC (-0.81 diopters vs. +0.75 diopters; p = 0.033).

ICG digital videoangiography was performed from the time the dye entered the fundus (0 seconds) to 30 seconds (Figs 1B, 1E, 2B, 2E, 3B, 3E, 4B and 4E). Choroidal perfusion showed pulsatile flow linked to the heart beat. Pulsatile dye filling was evident during the first 5 seconds. At 0 to 1 second, the choroidal arteries started to perfuse, and the dye filled the choriocapillaris at 1 to 2 seconds. The vortex veins were perfused from 2 to 5 seconds. By 20 seconds, all choroidal vessels were saturated with dye and the pulsatile flow was obscured.

Geographic filling delay areas were seen in all 32 eyes during perfusion of the choriocapillaris (Figs 1C, 2C, 3C and 4C) and even during the arterial phase (Figs 1B, 2B, 3B and 4B). These areas of filling delay were manually outlined in yellow (Figs 1I, 2I, 3I and 4I). The extent of filling delay was sharply demarcated in acute CSC (Figs 1I and 2I) but was obscure in chronic CSC (Figs 3I and 4I). Filling delay areas remained hypofluorescent during the venous phase even after 20 seconds in all eyes (Figs 1D, 1E, 2D, 2E, 3D, 3E, 4D and 4E).

The consistency of overlapping between the filling delay areas in choriocapillaris and the dilated vortex vein region in en-face images was grade 2.62 ± 0.49 in acute CSC (Figs 1K and 2K) and grade 1.55 ± 0.78 in chronic CSC (Figs 3K and 4K) in average (p = 0.0005). Acute CSC included 13 eyes of grade 3 and 8 eyes of grade 2. Chronic CSC included one eye of grade 3, 5 eyes of grade 2, 4 eyes of grade and one eye of grade 0.

In en-face images of Haller layer, asymmetry of upper and lower vortex veins was evident in 17 of 22 eyes (81%) with acute CSC and 6 of 11 eyes (54.5%) in chronic CSC (p = 0.114). In the remaining eyes, asymmetry was unclear because the border of both regions were ill defined.

Central choroidal thickness was significantly greater in eyes with acute CSC than in those with chronic CSC (411 ± 79 μm vs. 326 ± 64 μm; p = 0.004). In the posterior fundus with a 4500 μm diameter, for the areas which had been binarized to luminal and stromal areas, the ratio of the Haller layer area to total choroidal area was 63.7 ± 8.6% in acute CSC and 57.1 ± 7.9% in chronic CSC (p = 0.047). The ratio of the luminal area in the Haller layer area to total choroidal area was 46.9 ± 7.6% in acute CSC and 40.0 ± 6.9% in chronic CSC (p = 0.014).

The sites of dye leakage on FA images were always at the terminal areas of the dilated vortex veins in the en-face images (Figs 1J, 1K, 2J, 2K, 3J, 3K, 4J and 4K).

The characteristics of the 2 groups are summarized in Table 1.

**Table 1:** Patient demographic and clinical characteristics.

	Acute CSC	Chronic CSC	P Value
No. of eyes	21	11	
Age (yrs)	46.8 ± 8.9	61.8 ± 13.3	0.005
Male/Female	16/5	7/4	0.362
BCVA (logMAR)	0.09 ± 0.17	0.22 ± 0.33	0.286
Refraction (diopter)	-0.81 ± 1.76	+0.75 ± 1.91	0.033
Central choroidal thickness (μm)	411 ± 79	326 ± 64	0.004
Ratio of Haller layer area to total choroidal area (%)	63.7 ± 8.6	57.1 ± 7.9	0.047
Ratio of luminal area in Haller layer to total choroidal area (%)	46.9 ± 7.6	40.0 ± 6.9	0.014
Consistency of filling delay area and dilated vortex vein region (grade 3–0)	2.62 ± 0.49	1.55 ± 0.78	0.0005
Asymmetry of vortex vein	17 (81.0%)	6 (54.5%)	0.114
Leak point in FA over dilated vortex vein	21	11	

BCVA = best-corrected visual acuity; logMAR = logarithm of the minimum angle of resolution; FA = fluorescein angiography.

Grade 0: no tendancy of overlapping in both areas

Grade 1: less than 50% of filling delay area is overlapped in the dilated vortex vein region.

Grade 2: 50% or more of filling delay area is overlapped in the dilated vortex vein region.

Grade 3: filling delay area is all involved in dilated vortex vein region.

## Discussion

The current study showed geographic filling delay areas in the choriocapillaris on ICGA images to correspond well with the regions of the dilated dominant vortex veins in en-face images (Figs 1–4). Overlapping of the two areas was significantly higher (p = 0.0005) in acute CSC (consistency grade: 2.62 ± 0.49) than in chronic CSC (1.55 ± 0.78). These findings suggested that the blood flow into the choriocapillaris is blunted by congestion of the vortex veins supplying the areas that show filling delay.

Pachychoroid was more evident in acute than in chronic CSC. The mean central choroidal thickness was 411μm in acute CSC and 326 μm in chronic CSC. The mean ages of patients with acute and chronic CSC were 46.8 and 61.8 years, respectively. In healthy subjects in their 40s and 60s, central choroidal thicknesses, measured using SS-OCT, were reported to be 345μm and 208μm, respectively [23]. In the age-matched comparison, the choroid was found to be significantly thicker in our patients with acute and chronic CSC than in subjects with healthy eyes.

To quantify the vascular component in the choroid, Sonoda et al developed the binarization method [21]. This technique facilitates the distinction of luminal and stromal areas of the choroid in B-scan OCT images. Using this method, Argawal et al reported that eyes with acute CSC had a significantly higher choroidal vascularity index than their fellow eyes [24]. In the current study, we used the binarization method to evaluate the contribution of dilated vortex veins in the pachychoroid. The ratio of the Haller layer area to total choroidal area was 63.7 ± 8.6% in acute CSC and 57.1 ± 7.9% in chronic CSC (p = 0.047). The ratio of the luminal area in the Haller layer area to total choroidal area was 46.9 ± 7.6% in acute CSC and 40.0 ± 6.9% in chronic CSC (p = 0.014). This confirmed that dilatation of vortex veins is a major factor contributing to increased choroidal thickness in acute CSC. The lumens of vortex veins were attenuated in chronic CSC. Congestion of vortex veins may resolve in chronic CSC. Using laser speckle flowgraphy, Saito et al showed that macular choroidal blood flow velocity decreases concurrently with regression of CSC [25].

Asymmetry of dilated vortex veins was evident in 81% of acute CSC and 54.5% of chronic CSC cases (p = 0.114). In the current study, central choroidal thickness was decreased in chronic CSC and the ratio of the luminal area in the Haller layer to the total choroidal area was decreased in chronic CSC. These observations prompted us to ask why vortex vein asymmetry is lost in chronic CSC. We assume that collateral vessels form from the congested vortex region adjacent to the healthy quadrant of vortex veins, thereby obscuring the territory and the asymmetric dilatation of dominant vortex veins. We previously observed remodeling of the choroidal drainage route in eyes with occluded vortex veins, due to radiation choroidopathy [26] and scleral buckling for retinal detachment [27], using wide-angle ICGA. In the eyes with scleral buckling, choroidal veins were congested in the quadrant showing occluded vortex veins within 3 months after retinal detachment surgery. After 3 months, or longer in some cases, new drainage routes developed that connected the sector containing the occluded vortex veins to that of the intact vortex veins via venovenous anastomoses. Venous congestion showed resolution in the areas of the occluded vortex veins. Collateral veins formed between the superior and inferior vortex veins in 10 eyes and between the temporal and nasal vortex veins in two eyes. The theory of collateral vessel formation may explain the choroidal thickness decrease, vortex vein attenuation and the loss of vortex vein asymmetry in chronic CSC.

In the current study, we were not able to observe lobular patterns during the filling phase in the choriocapillaris on ICGA (Figs 1C, 2C, 3C and 4C). The lobular pattern of dye filling in the choriocapillaris was reported by Hayreh, studying FA images of monkey eyes with artificially elevated intraocular pressure [28]. He theorized that the choriocapillaris is comprised of many lobules in the posterior pole, each of which is an independent circulatory unit with a central arteriole and peripheral venule (central arteriole theory). We recently reproduced Hayreh’s experiment using fluorescein and ICG digital videoangiography (HRA) [29]. FA showed findings similar to his results. However, ICGA exhibited an inverse pattern. The dye first filled the outer frame of the lobule and then filled the lobule. The outer frame of lobules corresponded to the arteriolar network derived from the short posterior choroidal arteries. Blood perfused the lobules from the periphery to the center and then drained into the central venules (central venule theory). The lobular pattern seen on the FA images appeared to reflect the fluorescein which had extravasated through fenestrations in the choriocapillaris. The surrounding dark rim corresponded to the peripheral arterioles which have no fenestrations. We concluded that the choriocapillaris is not comprised of independent circulatory units but, rather, is perfused by branches of the short posterior arteries that form an arteriolar network at the level of the choriocapillaris [29]. Based on our experimental study, we believe that the arteriolar network supplies the blood flowing into the choriocapillaris and that the central venules within the network serves as the drainage route. Central venules are actually the terminal ends of vortex veins. Because the choroid has no tissue-capillary complex between the artery and the vein, the elevated venous pressure of the dilated vortex veins may directly affect the regional choriocapillaris via the central venules, which may account for the filling delay in ICGA. The elevated hydrostatic pressure may lead to breakdown of the barrier in the choriocapillaris or retinal pigment epithelium (RPE), resulting in serous retinal detachment. In this study, the sites of dye leakage in FA images were consistently located at the terminal areas of the dilated vortex veins (Figs 1J, 1L, 2J, 2L, 3J,3L, 4J and 4L).

What causes vortex vein congestion? We speculate that the cause is stenosis of the scleral channel. Proximal ends of the vortex veins form an ampulla at the equator, from which the vortex veins pass through the sclera. Although the sclera is 0.4 to 0.6 mm thick at the equator, vortex veins penetrate the sclera obliquely; thus, the length of the scleral channel is about 4 mm [30]. Eyes with a thick sclera seem to be vulnerable to obstruction. CSC does not develop in thin sclera as in eyes with high myopia but uveal effusion may occur in eyes with thick sclera. Many investigators have reported the efficacy of scleral window surgeries for uveal effusion [31–36]. In the current series, the mean refraction was -0.81 D in acute CSC and +0.75 D in chronic CSC. No eyes had high myopia or high hyperopia. If the causative lesion of vortex vein occlusion is the scleral channel, decompression of the vortex vein might be an effective treatment for CSC. Venkatesh et al. recently reported partial thickness scleral resection for chronic CSC in an eye with a short axial length and thick sclera [37]. Postoperatively, the exudative retinal detachment and RPE detachment resolved.

The current study had limitations. The en-face choroidal images were limited to 9 x 12 mm of the posterior pole. FA and ICGA were performed using HRA with a 30-degree field that corresponded to an approximately 9 x 9-mm square in the fundus. We observed dilatation of the asymmetrically dominant vortex veins in the posterior pole but were unable to visualize the proximal sites, as far as the ampulla. We manually outlined the filling delay area and dilated vortex vein region. This procedure inevitably involves subjective assessments.

In conclusion, the blood flow into the choriocapillaris is delayed as a result of congestion of the regional vortex veins supplying this geographic area. CSC is a disease that can be characterized as showing vortex vein congestion developing in eyes with asymmetrical vortex veins.

## Supporting information

S1 Table Statistical analysis. (XLSX)

## Abbreviations

CSC: central serous chorioretinopathy
FA: fluorescein angiography
ICGA: indocyanine green angiography
SS-OCT: swept-source optical coherence tomography
